# Large Language Models for Heterogeneous Data Mining in Liver Disease: Framework Development and Retrospective Validation Study

**DOI:** 10.2196/92921

**Published:** 2026-09-04

**Authors:** Haiping Zhang, Xinming Li, Kechi Fang, Yinxue Ma, Lijuan Li, Huiping Yan, Yanmin Liu, Yanhua Yu, Jing Wang

**Affiliations:** 1 Clinical Laboratory Center, Beijing Youan Hospital, Capital Medical University, Beijing, P R China; 2 Clinical Research Center for Autoimmune Liver Disease, Beijing Youan Hospital, Capital Medical University, Beijing, P R China; 3 State Key Laboratory of Cognitive Science and Mental Health, Institute of Psychology, Chinese Academy of Sciences, No. 16, Lincui Road, Chaoyang District, Beijing, 100101, P R China, 86 10-64855841; 4 Department of Psychology, University of Chinese Academy of Sciences, Beijing, P R China; 5 Second Department of Liver Disease Center, Beijing Youan Hospital, Capital Medical University, Beijing, P R China

**Keywords:** large language models, liver disease, clinical data mining, natural language processing, electronic medical records, LLMs

## Abstract

**Background:**

Differentiating among liver disease entities such as autoimmune liver disease (AILD), drug-induced liver injury (DILI), and chronic hepatitis B (CHB) remains clinically challenging due to overlapping clinical manifestations and nonspecific laboratory findings. Conventional machine learning (ML) approaches rely mainly on structured laboratory data, whereas free-text clinical reports and other heterogeneous electronic medical record data are often underused. Large language models (LLMs) may provide a strategy for encoding heterogeneous clinical information, yet their usefulness for liver disease classification remains insufficiently evaluated.

**Objective:**

This study aimed to evaluate the usefulness of LLM-derived embeddings for clinical data mining in liver disease and to determine whether integrating these embeddings with laboratory variables improves classification across broad disease categories and closely related subtypes.

**Methods:**

We retrospectively analyzed electronic medical record data from 7543 patients with nonoverlapping liver disease etiologies treated at Beijing Youan Hospital, Capital Medical University, between 2010 and 2025. Three LLMs (Qwen3, Huatuo-o1, and II-Medical) generated semantic embeddings from standardized clinical text, combining free-text examination reports, and structured clinical observations. Performance was assessed in a 3-class etiological task (AILD, DILI, and CHB) and a 4-class task further subclassifying AILD into autoimmune hepatitis and primary biliary cholangitis. We compared embedding-only models, LLM-integrated ML models, and an ML-only baseline using the same structured variable set and preprocessing pipeline, with lightweight natural language processing encoders and zero-shot LLM reasoning as additional comparators. Models were developed using 5-fold cross-validation and evaluated on an internal holdout set using accuracy, macroaveraged precision, recall, and *F*_1_-score.

**Results:**

In the 3-class task, the LLM-integrated ML models achieved macro *F*_1_-scores of 0.835‐0.837, compared with 0.791 for the ML-only baseline, with corresponding accuracies of 0.925‐0.929 versus 0.893. In the 4-class task, the LLM-integrated ML models achieved macro *F*_1_-scores of 0.717‐0.734, compared with 0.665 for the ML-only baseline, with corresponding accuracies of 0.920‐0.922 versus 0.874. A temporal split sensitivity analysis using cases from 2010 to 2019 for training and cases from 2020 to 2025 for testing showed that the relative advantage of LLM-integrated ML models over the ML-only baseline was preserved. Direct zero-shot LLM reasoning and lightweight natural language processing encoders performed below the embedding-based integrated models.

**Conclusions:**

In this single-center retrospective cohort of patients with clear-cut, nonoverlapping liver disease etiologies, LLM-derived embeddings provided complementary information to structured laboratory variables for multiclass liver disease classification. The integrated framework showed improved internal validation performance compared with the ML-only model, particularly for non-CHB categories and fine-grained subtype discrimination. Because patients with overlapping liver disease etiologies were excluded, the reported performance may overestimate diagnostic accuracy in broader real-world clinical settings where overlapping syndromes are common. Multicenter external validation and prospective evaluation in more heterogeneous patient populations are needed before clinical implementation.

## Introduction

Liver diseases span a wide spectrum of chronic conditions with diverse etiologies, and accurate diagnosis is crucial for appropriate treatment and improved prognosis [1,2]. Autoimmune hepatitis (AIH), primary biliary cholangitis (PBC), drug-induced liver injury (DILI), and chronic hepatitis B (CHB) differ significantly in their pathogenesis and clinical management [3-6]. However, these conditions often exhibit overlapping symptoms and laboratory indicators, posing a significant challenge for differential diagnosis in routine clinical settings [7,8]. Therefore, developing automated frameworks to assist in identifying liver disease subtypes is of substantial clinical importance [9-12].

The advent of modern hospital information systems has provided access to vast amounts of structured laboratory results and unstructured clinical narratives. However, these data are highly heterogeneous. Critical diagnostic information is often embedded in imaging and narrative reports, which are difficult to quantify using traditional statistical methods [13,14]. Furthermore, conventional machine learning (ML) pipelines rely heavily on manual feature engineering [15,16], such as outlier handling and variable selection [17], which are time-consuming and may fail to fully capture latent patterns within multisource clinical data.

Large language models (LLMs) have recently demonstrated exceptional capabilities in medical text understanding [18,19]. LLMs can act as general-purpose feature extractors, transforming unstructured clinical text into dense semantic embeddings. This facilitates the integration of narrative information with structured data and reduces the need for manual preprocessing [20]. Despite their potential, the use of LLM-based embeddings as feature encoding tools for downstream predictive modeling remains underexplored in liver disease research [21,22].

In this study, we developed an LLM-integrated framework for liver disease classification using heterogeneous electronic medical record (EMR) data. We hypothesized that semantic embeddings derived from free-text clinical reports and standardized clinical observations could capture diagnostic information not fully represented by structured laboratory variables alone. To test this hypothesis, we analyzed a real-world retrospective cohort of 7543 patients with liver disease treated between 2010 and 2025. We evaluated whether LLM-derived embeddings, alone or integrated with clinical laboratory variables, could improve classification across 2 diagnostic settings: broad etiological classification of autoimmune liver disease (AILD), DILI, and CHB, and fine-grained discrimination among AIH, PBC, DILI, and CHB. We further compared the integrated framework with ML-only models and exploratory direct zero-shot LLM reasoning.

## Methods

### Study Design

**Figure 1.**
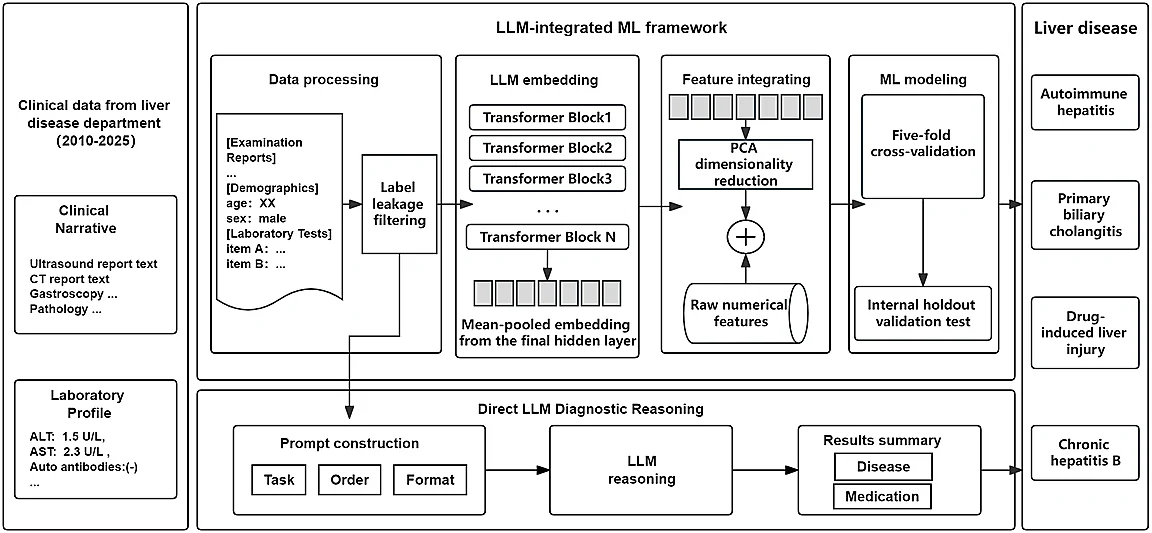
Overview of the study design and modeling framework. Electronic medical record data, including free-text clinical reports and structured laboratory variables, were extracted and converted into standardized patient-level inputs. Three LLM encoders generated semantic embeddings, which were integrated with clinical laboratory variables for downstream ML classification. Model development was performed using stratified 5-fold cross-validation, followed by final evaluation on an internal holdout validation set. A direct zero-shot LLM reasoning pathway was assessed in parallel for comparison. The final classification framework was evaluated across 4 liver disease categories (autoimmune hepatitis, primary biliary cholangitis, drug-induced liver injury, and chronic hepatitis B). ALT: alanine aminotransferase; AST: aspartate aminotransferase; CT: computed tomographic; LLM: large language model; ML: machine learning; PCA: principal component analysis.

The overall study design is illustrated in Figure 1. This was a retrospective framework development and internal validation study based on EMR data from a single tertiary liver disease center. Eligible patients were identified from the EMR system, and structured laboratory indicators and unstructured clinical narratives were extracted. LLM-based semantic embeddings were generated from standardized clinical text inputs and integrated with clinical laboratory variables. Classification models were then developed and evaluated against ML-only pipelines and direct LLM inference.

### Data Collection and Dataset Construction

We analyzed patient data retrieved from the EMR system of Beijing Youan Hospital, Capital Medical University. The initial screening included 162,159 patients seen between January 2010 and June 2025. The EMR contained structured laboratory test results and unstructured clinical narratives generated during routine clinical care, including imaging, endoscopy, and pathology-related reports, if available.

The study focused on patients diagnosed with CHB, DILI, and AILD. Within the AILD spectrum, AIH and PBC were included as the major subtypes. Patients with rarer autoimmune hepatobiliary diseases, including primary sclerosing cholangitis and IgG4-related sclerosing cholangitis, were excluded from the final analytical cohort because their limited sample sizes precluded robust statistical modeling.

### Inclusion and Exclusion Criteria

Inclusion criteria were (1) age ≥18 years, (2) at least 2 independent laboratory records supporting the same diagnosis, and (3) availability of medication and treatment records. Diagnostic labels were assigned according to established international guidelines supplemented by Chinese national guidelines. Specifically, AIH was defined according to the International Autoimmune Hepatitis Group simplified criteria [23], PBC according to the European Association for the Study of the Liver and Chinese Society of Hepatology guidelines [24,25], DILI according to the Roussel Uclaf Causality Assessment Method [26,27], and CHB according to the American Association for the Study of Liver Diseases along with the 2022 guidelines jointly issued by the Chinese Society of Hepatology and the Chinese Society of Infectious Diseases [28,29].

Because the enrollment period spanned from 2010 to 2025, all historical cases were retrospectively readjudicated by a senior hepatologist using archived clinical, serological, laboratory, imaging, and treatment data. Cases that no longer met the current criteria or lacked sufficient evidence for diagnostic confirmation were excluded to ensure label consistency across the 15-year study window.

Exclusion criteria were (1) coexistence of multiple competing liver etiologies, such as AIH-PBC overlap syndrome or CHB combined with DILI; (2) major comorbidities likely to independently affect liver biochemistry, lipid metabolism, or imaging findings, including malignant tumors and diabetes mellitus; (3) alcohol-related liver disease; (4) duplicate records for which only the first eligible encounter with a confirmed diagnosis was retained; and (5) patient-level missingness exceeding 40% across candidate clinical variables. These criteria were applied to reduce etiological ambiguity and ensure diagnostic certainty in this initial multiclass classification task involving nonoverlapping target etiologies. Patients with common metabolic comorbidities other than diabetes mellitus, including hypertension, dyslipidemia, obesity, and metabolic dysfunction–associated steatotic liver disease with no diabetes, were retained in the cohort to preserve generalizability to typical clinical populations.

### Dataset Partitioning

After applying the inclusion and exclusion criteria, the final cohort comprised 7543 patients across 3 disease categories, AILD (n=702), DILI (n=992), and CHB (n=5849). The AILD group consisted of AIH (n=256) and PBC (n=446). The cohort was stratified by the 4-class labels (AIH, PBC, DILI, and CHB) and randomly split into training (80%) and internal holdout validation (20%) sets. This stratification ensured class proportion preservation for both the 3-class task (where AIH and PBC are merged into AILD) and the 4-class task. Within the training set, we performed 5-fold cross-validation for dimensionality reduction and hyperparameter tuning [30]. The internal holdout validation set was kept separate from all model development procedures and was used only for final performance evaluation [31].

### Ethical Considerations

The study was approved by the Ethics Committee of Beijing Youan Hospital, Capital Medical University under protocol LL-2025‐029-K. The approved retrospective data use protocol covered the extraction and analysis of deidentified EMR data collected between January 2010 and June 2025. The requirement for informed consent was waived by the ethics committee. Data privacy was protected by deidentifying all records before analysis. All LLM encoding and reasoning tasks were performed locally to minimize the risk of information leakage.

### Construction of LLM Embedding and Integrated Features

Three 8B-parameter LLMs were selected as clinical data encoders, including Huatuo-o1 [32], II-Medical [33], and Qwen3 [34]. Huatuo-o1 and II-Medical are domain-specific medical LLMs, while Qwen3 is a general-purpose LLM. Exact repository identifiers and checkpoint versions for local deployment of each model are provided in Multimedia Appendix 1 to support computational reproducibility. To determine whether large medical LLMs provided incremental value over lighter text encoders, we also evaluated 2 lightweight natural language processing encoders, Doc2Vec [35] and BGE-M3 [36].

For each patient, structured and unstructured EMR contents were consolidated into a standardized modular text format. Examination items were organized as “<examination item> : <content>” and grouped into clinical modules, including demographics, laboratory tests, imaging reports, endoscopy reports, and pathology-related descriptions when available. To reduce potential label leakage before LLM encoding, explicit diagnostic labels, target disease names, discharge diagnoses, prescribed medication names, and other postdiagnostic information were removed or masked using predefined filtering rules [37]. These rules were applied to both structured medication fields and unstructured free-text narratives to identify and mask medication names and disease-specific treatment keywords, including highly diagnosis-informative terms such as specific antiviral agents and ursodeoxycholic acid. The complete list of high-risk keywords used for filtering is provided in Multimedia Appendix 2. A processed example is shown in Multimedia Appendix 3.

For each patient, dense embeddings of dimension 4096 were extracted by mean-pooling the token-level hidden states of the final transformer layer of each LLM, followed by L2 normalization, in a zero-shot setting, without any task-specific fine-tuning. To balance computational efficiency with information retention, principal component analysis (PCA) was applied to project the embeddings into lower-dimensional representations. Three target dimensions, 32, 64, and 128, were systematically evaluated. PCA fitting was performed only within the training data during cross-validation to prevent information leakage. The resulting LLM embeddings were then integrated with clinical laboratory variables to construct LLM-integrated feature sets. This integration strategy preserved interpretable clinical measurements while adding latent semantic representations derived from unstructured clinical narratives.

### Prompt Engineering and Direct LLM Reasoning

To explore the direct clinical reasoning capability of LLMs, we implemented a structured prompt engineering workflow. Models were prompted via a structured template that specified the role definition, task instructions, input constraints, and standardized output formats for disease classification and medication recommendation [38,39]. The prompt template is shown in Multimedia Appendix 4. All reasoning tasks were conducted in a zero-shot setting without task-specific fine-tuning [40].

To improve reproducibility, deterministic generation was used whenever supported by the model. The temperature was set to 0, and sampling was disabled by setting do_sample to false. Detailed inference hyperparameters, including maximum output length and decoding settings for each model, are provided in Multimedia Appendix 5. All LLM inference, including embedding extraction and zero-shot reasoning, was performed locally on a workstation equipped with 8 NVIDIA GeForce RTX 2080 Ti graphics processing units with 11 GB of video random access memory each.

Direct LLM diagnostic performance was evaluated by comparing the model-predicted disease category, parsed from the structured output field specified in the prompt template, with the reference diagnosis. Outputs that failed to match any of the predefined categories were classified as incorrect predictions.

Medication recommendation performance was assessed as an exploratory end point. Prescription records served as the reference list for comparison. To prevent label leakage, all medication-related content was removed from the input before it was supplied to the LLMs, and the models were prompted to generate medication recommendations from the remaining clinical narratives and laboratory data.

A medication match was defined as an exact or synonym-normalized match between a recommended drug class and a recorded medication class [41]. For each patient, medication precision was defined as the number of correctly recommended drug classes divided by the total number of recommended drug classes, and medication recall was defined as the number of correctly recommended drug classes divided by the total number of prescribed drug classes. Patient-level macroaveraged precision, recall, and *F*_1_-score were averaged across patients and reported as the primary medication recommendation metrics. When no medication was recommended, precision and *F*_1_-score were set to 0 for that patient. The any-match rate was retained only as a secondary descriptive indicator. Because actual prescriptions may reflect disease severity, contraindications, physician preference, drug availability, and temporal changes in treatment strategy, medication matching was interpreted as an exploratory indicator of clinical plausibility rather than a measure of treatment correctness.

### ML-Only Modeling

For comparative analysis, an ML-only pipeline was developed using structured clinical laboratory variables without LLM-derived embeddings [42]. To ensure a fair comparison with the LLM-integrated ML models, the ML-only model and the LLM-integrated ML models used the same structured clinical variable set and the same preprocessing pipeline. The only difference was that the LLM-integrated models additionally incorporated LLM-derived semantic embeddings.

For continuous variables, only those with at least 80% of nonmissing values (ie, <20% missingness) were retained, and missing values were imputed using the k-nearest neighbors algorithm, which estimates missing values based on intersample similarity among clinical variables. For categorical variables, categorical missingness itself may carry clinical information (eg, a test not being ordered); missing values were therefore encoded as a separate missing category rather than imputed to retain potentially informative missingness patterns in real-world EMRs. This missingness-handling scheme was applied specifically to the variable set used for model development and differs from the descriptive statistics reported, which summarize the originally observed (nonimputed) values.

All retained clinical variables were used directly for modeling without additional feature selection or dimensionality reduction. To prevent information leakage, imputation and scaling were fitted only on the training subset within each cross-validation fold and were then applied to the corresponding validation subset [43]. After hyperparameter selection, the complete preprocessing and modeling pipeline was refitted on the full development set and applied once to the internal holdout validation set for final performance evaluation.

### Model Training and Evaluation

Two classification tasks were implemented. The 3-class task classified patients as AILD, DILI, or CHB. The 4-class task classified patients as AIH, PBC, DILI, or CHB to evaluate fine-grained discrimination within AILD. Models were developed within the development set using stratified 5-fold cross-validation. Five downstream ML algorithms were evaluated in parallel, including random forest [44], multilayer perceptron (MLP) [45], logistic regression (LR) [46], Extreme Gradient Boosting (XGBoost) [47], and support vector machine (SVM) [48]. For each candidate hyperparameter configuration, model training and validation were performed across all 5-folds, and the mean validation macro *F*_1_-score was computed. The configuration with the highest mean validation macro *F*_1_-score was selected for final deployment. The hyperparameter search space and fixed training parameters are provided in Multimedia Appendix 6.

Class imbalance was addressed during model training. For random forest, LR, and SVM, class_weight = 'balanced' was used to assign weights inversely proportional to class frequencies. For MLP and XGBoost, equivalent sample-weighting strategies were applied during training. These strategies were used only within the training folds [49].

All preprocessing steps were incorporated into fold-specific training pipelines. For models requiring feature scaling, including MLP, LR, and SVM, *z* score normalization was fitted only on the training subset and then applied to the validation subset. PCA for LLM embeddings followed the same rule and was fitted only on the training subset within each fold. After cross-validation, the selected preprocessing steps and hyperparameter configuration were refitted on the full development set and evaluated once on the internal holdout validation set.

Model performance on the internal holdout validation set was assessed using accuracy, macro precision, macro recall, macro *F*_1_-score, and macroaveraged area under the receiver operating characteristic curve. Macroaveraged metrics were used to assign equal weight to each disease class and reduce bias caused by the predominance of CHB cases. All performance metrics are reported with 95% CIs computed by stratified bootstrap resampling with 1000 iterations on the internal holdout validation set, preserving class proportions across iterations. The ML algorithm achieving the highest cross-validation performance within the training set was selected as the representative model and subsequently evaluated on the internal holdout validation set for cross-strategy comparison.

As a sensitivity analysis, we also performed temporal internal validation using cases from 2010 to 2019 for training and cases from 2020 to 2025 for testing. Hyperparameter tuning was conducted only within the 2010‐2019 training set using 5-fold cross-validation, and the 2020‐2025 temporal test set was evaluated once.

### Statistical Analysis

Normality of continuous variables was assessed once in the pooled cohort using D’Agostino and Pearson omnibus test. Variables with fewer than 20 available observations were treated as non–normally distributed by default, given insufficient power to assess normality. Regardless of distribution, continuous variables were summarized as median (IQR) for the overall cohort and for each of the 4 diagnostic groups (AIH, PBC, DILI, and CHB), together with the number of patients with an available result in that specific column (N); group comparisons used 1-way ANOVA for variables identified as normally distributed in the pooled cohort and the Kruskal-Wallis test otherwise, and were not reported (shown as "—") when any of the 4 groups had fewer than 2 valid observations. Categorical variables were summarized as n/N (%) for the overall cohort and for each diagnostic group, where N is the number of patients with an available test result in that specific column rather than the corresponding column’s full sample size, since not all laboratory and serological tests were obtained in every patient; group comparisons used the chi-square test and were not reported when any expected cell frequency was below 5. For each variable, the number and percentage of patients without an available result in the full cohort (missing, n [%]) were additionally reported, using the full cohort size as the denominator. All statistical tests were 2-sided, and *P*<.05 was considered statistically significant. Statistical analyses were performed in Python (version 3.8; Python Software Foundation) using SciPy (version 1.7.3; SciPy Developers).

## Results

### Patients’ Clinical Characteristics

Baseline demographic and key clinical laboratory characteristics of the study cohort are summarized in Table 1. The internal holdout validation set exhibited distributions broadly comparable with those of the overall cohort, supporting its use for internal validation. Among the 7543 included patients, demographic features, biochemical profiles, viral hepatitis markers, autoimmune serological markers, and urinalysis findings varied across the 4 disease groups.

**Table 1.** Baseline demographic and clinical laboratory characteristics of the study cohort by disease group^a^.

Characteristic	Missing, n (%)	Total (N=7543)	AIH^b^ (n=256)	PBC^c^ (n=446)	DILI^d^ (n=992)	CHB^e^ (n=5849)	*P* value
Age (years), median (IQR)	0 (0.0)	44.0 (34.0-55.0), N=7543	54.0 (45.0-62.0), n=256	58.0 (50.0-65.0), n=446	50.0 (38.0-60.0), n=992	42.0 (32.0-52.0), n=5849	<.001
Male, n/N (%)	0 (0.0)	3924/7543 (52.0)	35/256 (13.7)	64/446 (14.3)	347/992 (35.0)	3478/5849 (59.5)	<.001
HBV^f^ DNA (+), n/N (%)	4792 (63.5)	1677/2751 (61.0)	0/10 (0.0)	1/18 (5.6)	1/57 (1.8)	1675/2666 (62.8)	—^g^
HBsAg^h^ (+), n/N (%)	4995 (66.2)	1739/2548 (68.2)	1/94 (1.1)	2/135 (1.5)	6/436 (1.4)	1730/1883 (91.9)	<.001
Anti-HBs^i^ (+), n/N (%)	1532 (20.3)	906/6011 (15.1)	55/123 (44.7)	93/204 (45.6)	257/579 (44.4)	501/5105 (9.8)	<.001
HBeAg^j^ (+), n/N (%)	1535 (20.3)	2059/6008 (34.3)	0/122 (0.0)	1/203 (0.5)	2/579 (0.3)	2056/5104 (40.3)	<.001
Anti-HBc^k^ (+), n/N (%)	1535 (20.3)	5346/6008 (89.0)	46/122 (37.7)	95/203 (46.8)	177/579 (30.6)	5028/5104 (98.5)	<.001
PreS1 Ag^l^ (+), n/N (%)	4977 (66.0)	1894/2566 (73.8)	0/18 (0.0)	0/48 (0.0)	3/100 (3.0)	1891/2400 (78.8)	—^g^
Quantitative HBsAg titer (IU/mL), median (IQR)	1377 (18.3)	496.1 (130.0-3603.0), N=6166	0.0 (0.0-0.0), n=191	0.0 (0.0-0.0), n=334	0.0 (0.0-0.0), n=753	774.1 (130.0-4105.3), n=4888	<.001
ALT^m^ (U/L), median (IQR)	4 (0.1)	40.0 (22.3-91.5), N=7539	50.0 (26.0-111.2), n=256	48.6 (24.1-83.0), n=444	106.3 (39.2-347.6), n=992	35.6 (21.0-73.0), n=5847	<.001
DBIL-to-TBIL^n^, median (IQR)	4 (0.1)	0.3 (0.2-0.4), N=7539	0.4 (0.3-0.6), n=256	0.4 (0.3-0.5), n=444	0.5 (0.3-0.7), n=992	0.3 (0.2-0.4), n=5847	<.001
GGT^o^ (U/L), median (IQR)	94 (1.2)	38.0 (19.1-94.0), N=7449	85.3 (44.7-176.1), n=253	154.2 (54.0-330.1), n=439	110.5 (54.8-222.7), n=955	29.5 (16.7-62.0), n=5802	<.001
ALP^p^ (U/L), median (IQR)	94 (1.2)	82.8 (63.5-115.0), N=7449	117.0 (85.0-177.0), n=253	169.0 (109.5-299.5), n=439	111.7 (82.2-159.1), n=955	76.0 (60.6-100.0), n=5802	<.001
Globulin (g/L), median (IQR)	4 (0.1)	29.2 (26.2-32.7), N=7539	34.2 (30.3-39.9), n=256	35.1 (30.9-40.5), n=444	28.9 (25.7-32.4), n=992	28.8 (26.1-32.0), n=5847	<.001
eGFR^q^ (mL/min/1.73m²), median (IQR)	566 (7.5)	111.7 (101.4-121.7), N=6977	105.6 (96.9-117.3), n=238	103.9 (95.0-111.6), n=429	109.1 (98.9-118.9), n=944	113.0 (103.1-122.7), n=5366	<.001
ASMA^r^ (+), n/N (%)	4656 (61.7)	130/2887 (4.5)	26/217 (12.0)	4/372 (1.1)	31/674 (4.6)	69/1624 (4.2)	<.001
ANA^s^ (+), n/N (%)	4607 (61.1)	1969/2936 (67.1)	210/217 (96.8)	322/373 (86.3)	487/683 (71.3)	950/1663 (57.1)	<.001
AMA^t^ (+), n/N (%)	4670 (61.9)	411/2873 (14.3)	43/212 (20.3)	301/369 (81.6)	42/669 (6.3)	25/1623 (1.5)	<.001
Anticytoskeleton (+), n/N (%)	4656 (61.7)	80/2887 (2.8)	20/217 (9.2)	4/372 (1.1)	17/674 (2.5)	39/1624 (2.4)	<.001
Antiparietal cell (+), n/N (%)	4656 (61.7)	148/2887 (5.1)	13/217 (6.0)	7/372 (1.9)	45/674 (6.7)	83/1624 (5.1)	.008
Bilirubin (urine) (+), n/N (%)	4464 (59.2)	482/3079 (15.7)	24/85 (28.2)	30/173 (17.3)	193/519 (37.2)	235/2302 (10.2)	<.001
Nitrite (urine) (+), n/N (%)	4464 (59.2)	77/3079 (2.5)	3/85 (3.5)	13/173 (7.5)	12/519 (2.3)	49/2302 (2.1)	—^g^
Ketone (urine) (+), n/N (%)	4464 (59.2)	169/3079 (5.5)	3/85 (3.5)	4/173 (2.3)	24/519 (4.6)	138/2302 (6.0)	—^g^

a Continuous variables are median (IQR), N; categorical variables are n/N (%). N is the number of patients with an available result in that specific column (total or subgroup) and may differ from the column header's nominal sample size. The “Missing, n (%)” column reflects overall cohort missingness only; subgroup-level completeness should be read from the N in each cell. Group comparisons used 1-way ANOVA or the Kruskal-Wallis test for continuous variables (per normality in the pooled cohort) and the chi-square test for categorical variables.

b AIH: autoimmune hepatitis.

c PBC: primary biliary cholangitis.

d DILI: drug-induced liver injury.

e CHB: chronic hepatitis B.

f HBV: hepatitis B virus.

g *P* values are not reported (“em dashes”) when test assumptions were not met or results would be uninformative.

h HBsAg: hepatitis B surface antigen.

i Anti-HBs: antibody to hepatitis B surface antigen.

j HBeAg: hepatitis B e antigen.

k Anti-HBc: antibody to hepatitis B core antigen.

l PreS1 Ag: pre-S1 antigen.

m ALT: alanine aminotransferase.

n DBIL-to-TBIL: direct bilirubin to total bilirubin ratio.

o GGT: γ-Glutamyl transferase.

p ALP: alkaline phosphatase.

q eGFR: estimated glomerular filtration rate.

r ASMA: antismooth muscle antibody.

s ANA: antinuclear antibody.

t AMA: antimitochondrial antibody.

Patients with PBC and AIH were older and predominantly female, with median ages of 58.0 and 54.0 years and male proportions of 14.3% and 13.7%, respectively. In contrast, patients with CHB were younger and predominantly male, with a median age of 42.0 years and a male proportion of 59.5%. Biochemical profiles varied in patterns consistent with the corresponding disease categories. Cholestatic markers were highest in patients with PBC, with median γ-glutamyl transferase and alkaline phosphatase values of 154.2 U/L and 169.0 U/L, respectively. Hepatocellular injury was most prominent in patients with DILI, with a median alanine aminotransferase of 106.3 U/L and the highest urinary bilirubin positivity rate (37.2%). Globulin levels were highest in patients with PBC (median 35.1 g/L), accompanied by lower estimated glomerular filtration rate values in both PBC and AIH compared with CHB, suggesting a degree of systemic involvement in autoimmune disease groups. Antimitochondrial antibody (AMA) positivity was frequent in PBC (81.6%), and antinuclear antibody (ANA) positivity was prevalent in both AIH and PBC (96.8% and 86.3%, respectively). Hepatitis B virus–related markers were concentrated in CHB, including hepatitis B surface antigen positivity of 91.9% and hepatitis B e antigen positivity of 40.3%. These serological and biochemical patterns were consistent with established diagnostic patterns [50-54] and supported the validity of the cohort for downstream modeling. Complete statistical results for all variables are provided in Multimedia Appendix 7.

### Feasibility of LLM-Based Embeddings in 3-Class Liver Disease Classification

To evaluate the discriminative potential of LLM-embedding models, we first conducted a 3-class task (AILD vs DILI vs CHB). As a reference, the ML-only model was developed using the full set of retained structured clinical laboratory variables without LLM-derived embeddings. This model achieved an accuracy of 0.893 (95% CI 0.877‐0.908) and a macro *F*_1_-score of 0.791 (95% CI 0.761‐0.820) on the internal holdout validation set.

We then evaluated LLM embedding-only models using LLM-derived semantic embeddings as stand-alone features. These models were trained without expert-defined text feature engineering or manually selected semantic variables. With a PCA dimensionality of 64, the Qwen3 embedding-only model achieved the highest accuracy of 0.919 (95% CI 0.907‐0.932), exceeding the ML-only model. The Huatuo-o1 and II-Medical embedding-only models achieved comparable performance, indicating that LLM-derived embeddings alone retained clinically relevant discriminative information from heterogeneous EMR inputs (Multimedia Appendix 8).

To contextualize the contribution of LLM-derived semantic representations, we compared their embedding-only models with 2 lightweight text representation methods. The 3 LLM embedding-only models outperformed Doc2Vec, which achieved an accuracy of 0.836 (95% CI 0.818‐0.854) and a macro *F*_1_-score of 0.635 (95% CI 0.596‐0.672), and BGE-M3, which achieved an accuracy of 0.838 (95% CI 0.819‐0.856) and a macro *F*_1_-score of 0.631 (95% CI 0.591‐0.669), on the internal holdout validation set (Multimedia Appendix 9). These findings suggest that LLM-derived embeddings provided more discriminative semantic information than the lightweight text encoders evaluated in this study.

Taken together, embedding-only analysis showed that LLM-derived representations could encode clinically relevant information from standardized EMR inputs and provide competitive performance in broad etiological liver disease classification. These results support their use as high-dimensional feature representations for downstream ML classification.

### Complementary Value of Integrated LLM and Clinical Features

**Figure 2.**
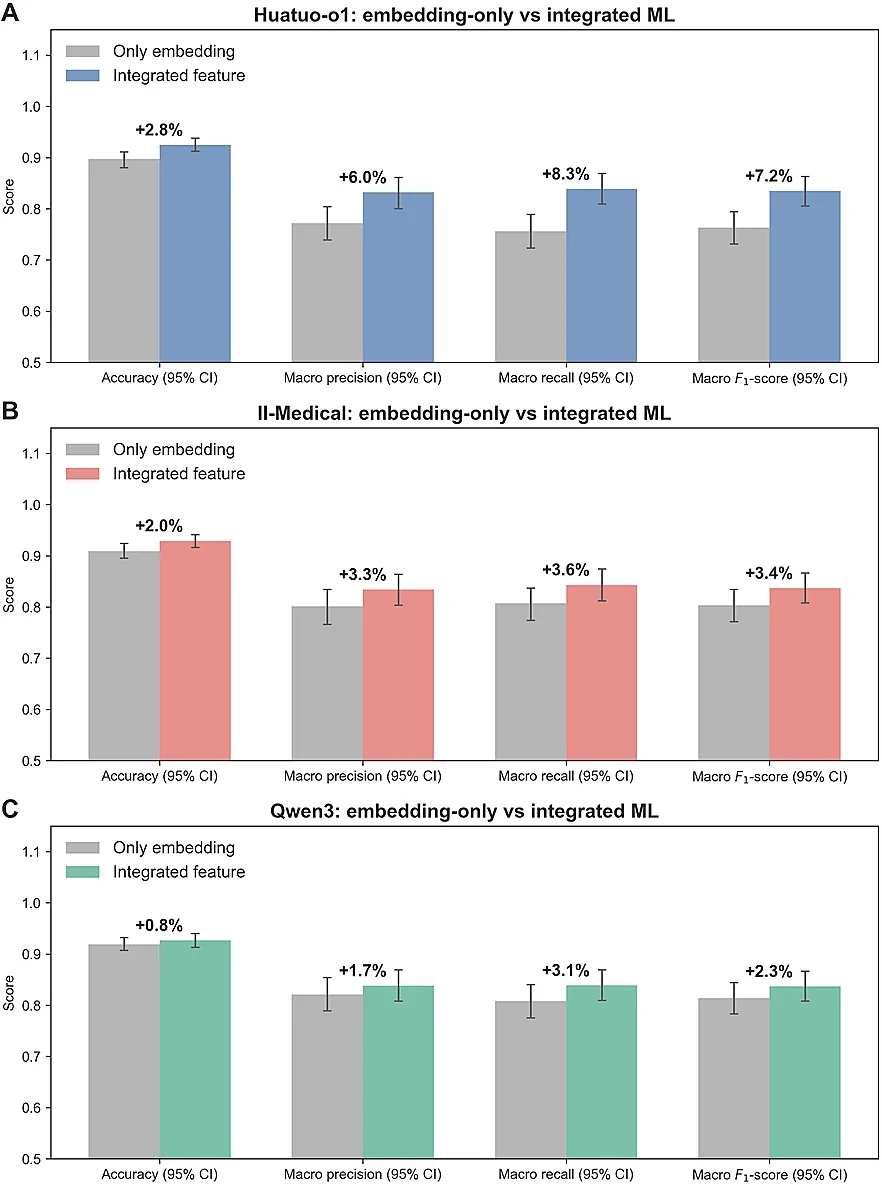
Three-class classification performance of large language model (LLM)–embedding-only and LLM-integrated ML models on the internal holdout validation set. The figure compares model performance in the 3-class task distinguishing autoimmune liver disease, drug-induced liver injury, and chronic hepatitis B. LLM embedding-only models used LLM-derived semantic embeddings as stand-alone features, whereas LLM-integrated ML models combined LLM-derived embeddings with clinical laboratory variables. (A) Performance comparison based on Huatuo-o1 embeddings, (B) performance comparison based on II-Medical embeddings, and (C) performance comparison based on Qwen3 embeddings. Across the evaluated LLMs, the integrated models showed higher accuracy, macro precision, macro recall, and macro *F*_1_-score than the corresponding embedding-only models. ML: machine learning.

We next evaluated whether LLM-derived embeddings provided complementary information when combined with structured clinical laboratory variables. After feature integration, the LLM-integrated ML models achieved accuracies ranging from 0.925 to 0.929 on the internal holdout validation set, corresponding to improvements of 0.8-2.8 percentage points over the respective LLM embedding-only models (Figures 2A-2C). All 3 LLM-integrated ML models also outperformed the ML-only baseline, with accuracy increasing by 3.2-3.6 percentage points and macro *F*_1_-score increasing by 4.4-4.6 percentage points (Table 2). Although the II-Medical–integrated ML model achieved the highest accuracy of 0.929 (95% CI 0.916‐0.941), the 3 LLM-integrated models showed comparable overall performance, with overlapping 95% CIs across the reported metrics.

**Table 2.** Three-class classification performance of ML^a^-only and LLM^b^-integrated ML models on the internal holdout validation set^c^.

Model	Accuracy (95% CI)	Macro precision (95% CI)	Macro recall (95% CI)	Macro *F*_1_-score (95% CI)
ML-only model	0.893 (0.877‐0.908)	0.766 (0.734‐0.797)	0.829 (0.799‐0.858)	0.791 (0.761‐0.820)
Huatuo-o1–integrated ML model	0.925 (0.912‐0.938)	0.832 (0.800‐0.861)	0.839 (0.809‐0.869)	0.835 (0.805‐0.863)
II-Medical–integrated ML model	0.929 (0.916‐0.941)	0.834 (0.803‐0.864)	0.843 (0.812‐0.874)	0.837 (0.808‐0.866)
Qwen3-integrated ML model	0.927 (0.913‐0.940)	0.838 (0.808‐0.869)	0.839 (0.809‐0.869)	0.837 (0.808‐0.866)

a ML: machine learning.

b LLM: large language model.

c The ML-only model used the full set of clinical laboratory variables without feature selection, and the LLM-integrated ML models combined LLM embeddings with the same full set of clinical variables. Macroaveraged metrics were computed by averaging each metric across classes, treating all classes equally regardless of prevalence.

Class-specific precision analysis showed that performance differences were more evident for AILD and DILI than for CHB (Table 3). AILD precision was 0.700 (95% CI 0.623‐0.777) in the ML-only model and ranged from 0.775 to 0.796 in the LLM-integrated ML models. DILI precision was 0.606 (95% CI 0.550‐0.661) in the ML-only model and ranged from 0.738 to 0.744 in the LLM-integrated ML models. In contrast, CHB precision remained high across all models, ranging from 0.977 to 0.991.

**Table 3.** Class-specific precision of ML^a^-only and LLM^b^-integrated ML models in the 3-class classification task on the internal holdout validation set.

Model	AILD^c^ precision (95% CI)	DILI^d^ precision (95% CI)	CHB^e^ precision (95% CI)
ML-only model	0.700 (0.623‐0.777)	0.606 (0.550‐0.661)	0.991 (0.985‐0.996)
Huatuo-o1–integrated ML model	0.775 (0.702‐0.846)	0.744 (0.684‐0.798)	0.977 (0.968‐0.985)
II-Medical–integrated ML model	0.780 (0.706‐0.849)	0.738 (0.683‐0.798)	0.983 (0.974‐0.990)
Qwen3-integrated ML model	0.796 (0.722‐0.866)	0.741 (0.684‐0.799)	0.977 (0.967‐0.985)

a ML: machine learning.

b LLM: large language model.

c AILD: autoimmune liver disease.

d DILI: drug-induced liver injury.

e CHB: chronic hepatitis B.

We further assessed the computational cost of LLM-integrated ML models. Per-patient end-to-end inference time, including tokenization, LLM embedding extraction, dimensionality reduction, feature concatenation, and classifier prediction, was longer than that of the ML-only model but remained approximately 670‐685 ms per case in the local benchmark (Multimedia Appendix 10). All models were deployed locally on graphics processing units, and the embedding-based pipeline used a single forward pass without autoregressive token decoding. This design explains the lower latency compared with API-based generative reasoning. These findings suggest that LLM-derived embeddings provide information complementary to structured clinical laboratory variables in the 3-class task. The contribution was most apparent for AILD and DILI, whereas CHB classification remained robust across modeling approaches.

### Performance in Refined 4-Class Classification Tasks

We next evaluated the feature integration strategy in a more refined 4-class classification task (AIH vs PBC vs DILI vs CHB) to assess whether LLM embeddings could further discriminate between clinically related AILD subtypes. The ML-only model achieved an accuracy of 0.874 (95% CI 0.857‐0.889) and a macro *F*_1_-score of 0.665 (95% CI 0.626‐0.701) on the internal holdout validation set. In comparison, the 3 LLM-integrated ML models achieved accuracies ranging from 0.920 to 0.922, corresponding to improvements of 4.6-4.8 percentage points over the ML-only model (Table 4). Macro *F*_1_-scores also increased, ranging from 0.717 to 0.734 across the LLM-integrated models. The Huatuo-o1–integrated ML model achieved the highest macro *F*_1_-score of 0.734 (95% CI 0.690‐0.774), while the 3 LLM-integrated ML models showed broadly comparable performance with overlapping 95% CIs across the reported metrics.

**Table 4.** Four-class classification performance of ML^a^-only and LLM^b^-integrated ML models on the internal holdout validation set.

Model	Accuracy (95% CI)	Macro precision (95% CI)	Macro recall (95% CI)	Macro *F*_1_-score (95% CI)
ML-only model	0.874 (0.857‐0.889)	0.635 (0.599‐0.672)	0.707 (0.664‐0.750)	0.665 (0.626‐0.701)
Huatuo-o1–integrated ML model	0.922 (0.907‐0.935)	0.750 (0.701‐0.800)	0.730 (0.691‐0.769)	0.734 (0.690‐0.774)
II-Medical–integrated ML model	0.920 (0.907‐0.934)	0.735 (0.686‐0.787)	0.717 (0.680‐0.757)	0.717 (0.677‐0.759)
Qwen3-integrated ML model	0.921 (0.907‐0.934)	0.746 (0.695‐0.802)	0.725 (0.690‐0.765)	0.727 (0.685‐0.769)

a ML: machine learning.

b LLM: large language model.

The LLM-integrated ML models also showed more balanced discrimination across disease classes, with higher macroaveraged area under the receiver operating characteristic curves than the ML-only model in the 4-class task (Multimedia Appendix 11). These findings suggest that LLM-derived embeddings provided complementary information for the refined 4-class task rather than improving performance only in the majority CHB category.

**Figure 3.**
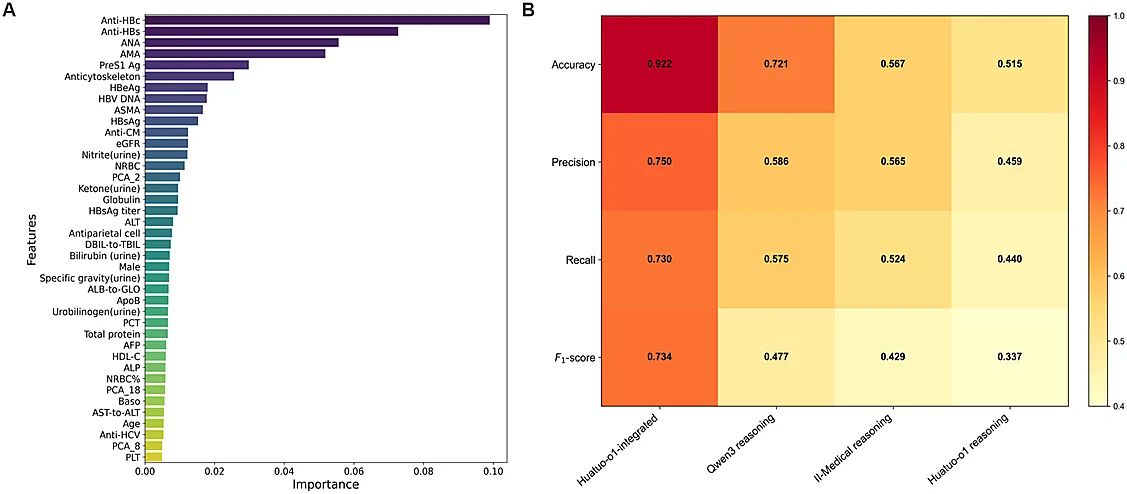
Feature importance of the large language model (LLM)–integrated machine learning (ML) model and comparison with direct LLM reasoning. (A) Top 40 features ranked by importance in the Huatuo-o1–integrated ML model. Conventional clinical variables, including viral hepatitis markers, autoantibodies, and liver function indicators, ranked among the most informative features. Several LLM-derived principal components, including PCA_2, PCA_18, and PCA_8, were also retained among the top-ranked features. (B) Performance comparison between the Huatuo-o1–integrated ML model and direct zero-shot reasoning by the 3 LLMs. The integrated ML model showed substantially higher accuracy (0.922 vs 0.515-0.721), macro precision (0.750 vs 0.459-0.586), macro recall (0.730 vs 0.440-0.575), and macro *F*_1_-score (0.734 vs 0.337-0.477) compared with direct LLM reasoning across all 3 models. AFP: alpha-fetoprotein; ALB-to-GLO: albumin to globulin ratio; ALP: alkaline phosphatase; ALT: alanine aminotransferase; AMA: antimitochondrial antibody; ANA: antinuclear antibody; Anti-CM: anticardiac muscle antibody; Anti-HBc: antibody to hepatitis B core antigen; Anti-HBs: antibody to hepatitis B surface antigen; ApoB: apolipoprotein B; ASMA: antismooth muscle antibody; AST: aspartate aminotransferase; DBIL-to-TBIL: direct bilirubin to total bilirubin ratio; eGFR: estimated glomerular filtration rate; HBeAg: hepatitis B e antigen; HBsAg: hepatitis B surface antigen; HBV: hepatitis B virus; HCV: hepatitis C virus; HDL-C: high-density lipoprotein cholesterol; NRBC: nucleated red blood cells; PCA: principal component analysis; PCT: plateletcrit; PLT: platelet count; PreS1 Ag: pre-S1 antigen.

Feature importance analysis identified both conventional clinical variables and LLM-derived components among the most informative features. Autoantibody markers, including AMA and ANA, remained highly ranked, consistent with their established relevance in AILD classification [55]. Several LLM-derived principal components, including PCA_2, PCA_18, and PCA_8, were also among the top-ranked features (Figure 3A). These components represent orthogonal dimensions of the compressed embedding space and should be interpreted as semantic representation features rather than directly interpretable clinical variables.

Together, these findings support the complementary value of LLM-derived representations when integrated with structured clinical variables for fine-grained liver disease classification.

### Temporal Split Sensitivity Analysis

To assess model stability under temporal evaluation, we performed a sensitivity analysis using cases from 2010 to 2019 for training and cases from 2020 to 2025 for testing. Hyperparameter tuning was performed only within the 2010‐2019 training set, and the 2020‐2025 temporal test set was evaluated once.

In the 3-class task, the LLM-integrated ML models maintained higher macro *F*_1_-scores than the ML-only baseline under temporal evaluation, with macro *F*_1_-scores ranging from 0.852 to 0.858 compared with 0.799 for the ML-only model. In the 4-class task, the same pattern was observed, with macro *F*_1_-scores ranging from 0.743 to 0.752 for the LLM-integrated ML models compared with 0.702 for the ML-only model. These findings indicate that the relative advantage of LLM-derived embeddings over the ML-only baseline was preserved under temporal internal validation. Full temporal split results are provided in Multimedia Appendix 12.

### Exploratory Assessment of Direct LLM Reasoning

Beyond the embedding-based integration framework, we examined whether LLMs could directly generate disease classification and medication recommendations through structured prompts. In direct diagnostic inference, Qwen3 achieved the highest performance among the 3 evaluated LLMs, with an accuracy of 0.721 and a macro *F*_1_-score of 0.477. However, this performance remained lower than that of the LLM-integrated ML framework. For example, the Huatuo-o1–integrated ML model achieved an accuracy of 0.922 and a macro *F*_1_-score of 0.734 in the 4-class task (Figure 3B).

Medication recommendation was evaluated as an exploratory end point by comparing model-generated drug classes with recorded prescriptions after medication name normalization. Agreement with actual prescriptions varied across models. II-Medical achieved the highest any-match rate of 57.5%, followed by Qwen3 at 52.6% and Huatuo-o1 at 18.9% (Multimedia Appendix 13). The same pattern was observed using list-level metrics. Patient-level macro precision was 0.542 for II-Medical, 0.493 for Qwen3, and 0.184 for Huatuo-o1. Patient-level macro *F*_1_-score was 0.280 for II-Medical, 0.249 for Qwen3, and 0.088 for Huatuo-o1.

The low recommendation rate observed for Huatuo-o1 was partly related to its tendency to recommend observation or no specific treatment for a substantial proportion of patients with CHB. This may represent clinically appropriate judgment but results in low overlap with recorded prescriptions under the current evaluation framework.

Overall, direct zero-shot LLM reasoning showed limited stand-alone diagnostic and medication recommendation performance compared with the embedding-based integration framework. These findings support the use of LLMs primarily as feature encoders within a controlled ML pipeline rather than as stand-alone diagnostic or therapeutic decision systems.

## Discussion

### Principal Findings

In this single-center retrospective study, we developed an LLM-integrated framework for multiclass liver disease classification using heterogeneous EMR data. The framework combined semantic embeddings derived from free-text clinical reports and standardized clinical observations with structured laboratory variables. On the internal holdout validation set, the integrated models achieved higher performance than the ML-only model in both the 3-class task and the more granular 4-class task. The improvement was most evident for non-CHB categories and for fine-grained classification involving AIH and PBC, 2 AILD subtypes with overlapping biochemical profiles but different clinical management pathways.

A central finding of this study is that LLM-derived embeddings may provide information complementary to conventional laboratory variables. Clinical laboratory markers remain essential for liver disease classification, particularly viral hepatitis markers and autoantibodies such as ANA and AMA. However, free-text examination reports and other clinical narratives may contain contextual information that is difficult to represent through predefined structured variables alone. By encoding these heterogeneous inputs into high-dimensional representations, the LLM-integrated framework provided a way to incorporate narrative information into downstream ML models without requiring extensive rule-based text structuring.

The analysis also supports the value of evaluating models with metrics appropriate for imbalanced multiclass data. Because CHB represented the largest disease group, overall accuracy alone could overemphasize performance in the majority class. We therefore reported macroaveraged precision, recall, and *F*_1_-score, together with class-specific results. These analyses showed that the LLM-integrated models improved macrolevel performance and performed better in non-CHB categories than the ML-only model. This pattern suggests that LLM-derived embeddings contributed most to disease categories with greater clinical heterogeneity rather than simply improving recognition of the dominant CHB group.

Feature importance analyses provided additional support for the complementary role of LLM-derived features. Conventional markers, including viral hepatitis markers, autoantibodies, and liver function indicators, remained among the most informative features. Several LLM-derived principal components were also ranked among the top features, suggesting that the embedding space retained discriminative information not fully captured by structured variables. However, these principal components should be interpreted as compressed semantic representations rather than as directly interpretable clinical concepts.

We also evaluated zero-shot LLM reasoning for liver disease classification and medication recommendation. Although this analysis showed exploratory value, direct LLM reasoning was less stable and less accurate than the embedding-based ML framework, and results should be interpreted with caution given the known sensitivity of zero-shot performance to prompt phrasing and instruction-tuning differences across models. Medication recommendation overlap should also be interpreted cautiously because prescriptions depend on disease severity, contraindications, patient history, and physician judgment. These findings support the use of LLMs as feature encoders within a controlled modeling pipeline rather than stand-alone diagnostic or therapeutic decision systems.

### Comparison With Prior Work

Previous liver disease prediction studies have often relied on selected biochemical markers, serological indicators, or quantitative imaging features [56,57]. These approaches remain clinically meaningful but may not fully capture information contained in free-text reports and other semistructured clinical records. Recent studies have begun to explore LLMs for clinical text understanding, information extraction, and diagnostic reasoning, but their role as embedding-based feature encoders for liver disease classification remains insufficiently studied [58,59].

Our study extends this work by evaluating LLM-derived embeddings in a real-world liver disease cohort and by comparing several modeling strategies, including ML-only models, LLM embedding-only models, LLM-integrated ML models, lightweight natural language processing baselines, and direct zero-shot LLM reasoning. This comparative design helps distinguish the contribution of semantic representation learning from that of downstream ML classifiers. The results suggest that LLM-derived embeddings can improve internal classification performance when integrated with structured clinical variables, especially in diagnostically challenging categories.

### Limitations

This study has several limitations. First, this was a single-center retrospective study, and primary evaluation used an internal holdout validation set rather than an external cohort. Although historical cases were retrospectively readjudicated using updated diagnostic criteria, multicenter external validation is still needed to assess generalizability across hospitals, laboratory systems, and documentation styles. A temporal internal validation using cases from 2010 to 2019 for training and cases from 2020 to 2025 for testing showed that the relative advantage of the LLM-integrated models over the ML-only baseline was preserved (Multimedia Appendix 12). However, this analysis remains an internal validation and does not substitute for external validation across independent centers. Second, predefined exclusions were applied to reduce etiological ambiguity, including the exclusion of patients with multiple coexisting liver disease etiologies and diabetes mellitus. Therefore, whether the framework generalizes to patients with overlapping liver disease mechanisms, substantial metabolic comorbidities, or metabolic dysfunction–associated steatotic liver disease as an independent diagnostic category requires further validation. Third, although missingness thresholds and sensitivity analyses were applied, residual bias from incomplete retrospective EMR data and imputation cannot be fully excluded. In particular, missingness in serological markers such as hepatitis B surface antigen, ANA, and AMA reflects selective test ordering rather than random unavailability. The missing-indicator code used for these variables may therefore partly capture this ordering pattern rather than a purely biological signal, and disentangling the 2 would require prospective or external validation. Fourth, although explicit diagnostic labels, prescribed medication names, and disease-specific treatment keywords were removed or masked before LLM encoding, narrative mentions of specific treatments embedded in free-text reports may not have been completely captured by the rule-based filtering process, representing a potential source of residual label leakage. Fifth, due to hardware constraints, only 8B-parameter LLMs were evaluated, and the impact of larger models or domain-specific fine-tuning on representation quality remains unknown. Sixth, PCA-compressed embedding features cannot be directly mapped to individual clinical concepts, limiting interpretability. Finally, the zero-shot reasoning and medication recommendation analyses were exploratory. Zero-shot performance is sensitive to prompt phrasing, and the use of a single fixed template may have favored certain models. Medication matching was based on prescription overlap and should not be interpreted as evidence of treatment appropriateness or clinical usefulness. Prospective workflow-based evaluation is needed before clinical implementation.

### Conclusions

In this single-center retrospective cohort, LLM-derived embeddings provided complementary representations to structured laboratory data for multiclass liver disease classification. The LLM-integrated framework showed improved internal validation performance compared with ML-only models, particularly for non-CHB categories and fine-grained subtype discrimination involving AIH and PBC. These findings support further evaluation of LLM-derived embeddings as part of structured clinical prediction pipelines, but multicenter external validation and prospective assessment are required before integration into clinical workflows.

### Future Work

Future work should evaluate this framework in multicenter cohorts with different laboratory systems, documentation styles, and disease distributions. Prospective studies are also needed to determine whether the model can improve diagnostic workflow, clinician agreement, or subsequent test ordering in real-world settings. Additional work should focus on improving the interpretability of LLM-derived embeddings and assessing whether similar approaches are useful in other clinical domains where free-text reports provide information complementary to structured laboratory data.

## Supplementary material

10.2196/92921 Multimedia Appendix 1 This supplementary material summarizes the basic information of three 8B-parameter large language models (Huatuo-o1, II-Medical, and Qwen3), including parameter size, release date, model type, maximum context length, underlying architecture, typical use cases, and the exact repository identifier and checkpoint or version tag used for local deployment.

10.2196/92921 Multimedia Appendix 2 This supplementary material provides the list of high-risk keywords that may result in label leakage.

10.2196/92921 Multimedia Appendix 3 This supplementary material provides an illustrative example of the standardized clinical text input used for large language model encoding.

10.2196/92921 Multimedia Appendix 4 This supplementary material shows a structured prompt template that assigns the large language model a hepatology assistant role and defines standardized clinical inputs and output format for differentiating liver disease.

10.2196/92921 Multimedia Appendix 5 This supplementary material contains the parameters for large language model inference.

10.2196/92921 Multimedia Appendix 6 This supplementary material includes 5-fold cross-validation grid search parameters.

10.2196/92921 Multimedia Appendix 7 This supplementary material contains the baseline demographics and comprehensive clinical laboratory characteristics of the study cohort.

10.2196/92921 Multimedia Appendix 8 This supplementary material shows the 3-class classification performance of the machine learning (ML)-only model and large language model (LLM)–embedding models on the internal holdout validation set. The gray line denotes the ML-only model using the full set of clinical laboratory variables, while colored bars denote LLM-embedding models using embeddings from Huatuo-o1 (blue), II-Medical (red), and Qwen3 (green). The Qwen3-embedding model reached an accuracy of 0.919 (95% CI 0.907-0.932).

10.2196/92921 Multimedia Appendix 9 This supplementary material includes the comparison of large language model embeddings and 2 lightweight natural language models for 3-class text classification.

10.2196/92921 Multimedia Appendix 10 This supplementary material reports the per-case end-to-end inference time of the machine learning (ML)–only and large language model (LLM)–integrated ML models, decomposed by pipeline stage (feature loading, tokenization, LLM-embedding extraction, dimensionality reduction, feature concatenation, and downstream classification). Timing was measured on 1000 randomly selected patients from the modeling dataset; values are reported in milliseconds per case as median, mean, SD, minimum, and maximum.

10.2196/92921 Multimedia Appendix 11 This supplementary material shows the receiver operating characteristic curves for 4-class liver disease classification of the machine learning (ML)–only and large language model (LLM)–integrated ML models on the internal holdout validation set. The 3 LLM-integrated models (Huatuo-o1, II-Medical, and Qwen3) achieved macroaverage area under the receiver operating characteristic curves (AUROCs) of 0.969-0.973, compared with 0.954 for the ML-only model. Autoimmune hepatitis discrimination improved (AUROC 0.922-0.933 vs 0.895), and class-wise AUROC varied less across diseases among the LLM-integrated models.

10.2196/92921 Multimedia Appendix 12 This supplementary material shows the 3-class and 4-class classification performance of the machine learning (ML)–only and large language model–integrated ML models under a temporal split sensitivity analysis, using cases diagnosed between 2010 and 2019 for training and cases diagnosed between 2020 and 2025 for testing.

10.2196/92921 Multimedia Appendix 13 This supplementary material shows the medication recommendation performance metrics across large language models.

## Abbreviations

AIH: autoimmune hepatitis
AILD: autoimmune liver disease
AMA: antimitochondrial antibody
ANA: antinuclear antibody
CHB: chronic hepatitis B
DILI: drug-induced liver injury
EMR: electronic medical record
LLM: large language model
LR: logistic regression
ML: machine learning
MLP: multilayer perceptron
PBC: primary biliary cholangitis
PCA: principal component analysis
SVM: support vector machine
XGBoost: Extreme Gradient Boosting
