# Supplementary material for: Large Language Models for Heterogeneous Data Mining in Liver Disease: Framework Development and Retrospective Validation Study
Source: J Med Internet Res. 2026 Sep 4;28:e92921. doi: 10.2196/92921 (PMC13544415; doi:10.2196/92921)
Supplement: Supplementary file 1 [file jmir-v28-e92921-s003.docx]

[Examination Reports]

Ultrasound findings:

Diffuse liver disease; cirrhosis cannot be excluded. Splenomegaly, dilatation of the portal and splenic veins, hepatic cyst, gallstones, multiple cholesterol deposits in the gallbladder wall, cholecystitis, and small-volume ascites were reported. No bilateral pleural effusion was detected

CT findings:

The thoracic cage was symmetrical bilaterally, and lung markings were clear. A slightly high-density sub-pleural nodular approximately 2 mm in diameter was observed in the left lower lobe. The trachea and major bronchi were patent. No obvious abnormalities were observed in the heart or great vessels. No pleural effusion or obviously enlarged mediastinal lymph nodes were detected.

Gastroscopy findings:

Severe esophageal varices with red color signs, type 1 gastric varices, severe portal hypertensive gastropathy, acute erosive hemorrhagic gastritis with bile reflux, and multiple duodenal bulb ulcers at the H1 stage were reported.

Radiology findings:

No significant abnormalities of the heart, lungs, or diaphragm were reported.

[Demographics]

Age: 48.0 years;

Sex: Male;

[Laboratory Tests]

Aspartate aminotransferase:38 U/L;

direct/total bilirubin ratio:0.31;

AST/ALT ratio:1.58;

total bilirubin:64.5 μmol/L;

alanine aminotransferase:24 U/L;

albumin:35.8 g/L;

direct bilirubin:19.9 μmol/L;

total protein:53.3 g/L;

globulin:17.5 g/L;

albumin/globulin ratio:2.05;

alkaline phosphatase:169.2 U/L;

gamma-glutamyl transpeptidase:44.7 U/L;

total bile acids:47.6 μmol/L;

platelet count:48 ×10⁹/L;

Anti-HBs antibody:<2.00 mIU/mL negative (-);

anti-HBe antibody: negative (-);

anti-HBc antibody: negative (-);

HBeAg: negative (-);

HBsAg: negative (-);

AFP:4.94 ng/mL;…;

antinuclear antibody:negative (-);

anti-smooth muscle antibody:negative (-);

anti-mitochondrial antibody:negative (-);

anti-hepatitis C virus antibody:negative (-);

APTT ratio:1.59;

prothrombin ratio:1.46;

prothrombin time:15.9 s;

fibrinogen:1.82 g/L;

INR:1.48;

…;

prothrombin activity:54%;

[Preprocessing Note]

Explicit diagnostic labels, discharge diagnoses, medication records, postdiagnostic notes, pathology conclusions with direct diagnostic implications, and high-risk disease- or treatment-related keywords were removed or masked before LLM encoding.
